# Progressing our understanding of the impacts of nutrition on the brain and behaviour in anorexia nervosa: a tyrosine case study example

**DOI:** 10.1186/s40337-021-00439-z

**Published:** 2021-07-13

**Authors:** Melissa Hart, David Sibbritt, Lauren T. Williams, Kenneth P. Nunn, Bridget Wilcken

**Affiliations:** 1 University of Newcastle, Callaghan, NSW 2308 Australia; 2 Hunter New England Mental Health Service, Waratah, NSW 2298 Australia; 3 University of Technology, Ultimo, NSW 2007 Australia; 4 Menzies Health Institute of Queensland, Griffith University, Southport, QLD 4125 Australia; 5 University of Sydney, Camperdown, NSW 2006 Australia

**Keywords:** Anorexia nervosa, Noradrenaline, Pharmacology, Tyrosine, case study

## Abstract

Anorexia nervosa is a severe and complex illness associated with a lack of efficacious treatment. The effects of nutrition on the brain and behaviour is of particular interest, though an area of limited research. Tyrosine, a non-essential amino acid, is a precursor to the catecholamines dopamine, noradrenaline and adrenaline. Ongoing tyrosine administration has been proposed as an adjunct treatment through increasing blood tyrosine sufficiently to facilitate brain catecholamine synthesis. The effects of tyrosine supplementation in adolescents with anorexia nervosa remain to be tested. This study had approval from the Hunter New England Human Research Ethics Committee (06/05/24/3.06). We aimed to explore the pharmacokinetics of tyrosine loading in adolescents with anorexia nervosa (*n* = 2) and healthy peers (*n* = 2). The first stage of the study explored the pharmacological response to a single, oral tyrosine load in adolescents (aged 12–15 years) with anorexia nervosa and healthy peers. Participants with anorexia nervosa then continued tyrosine twice daily for 12 weeks. There were no measured side effects. Peak tyrosine levels occurred at approximately two to three hours and approached baseline levels by eight hours. Variation in blood tyrosine response was observed and warrants further exploration, along with potential effects of continued tyrosine administration in anorexia nervosa.

**Supplementary Information:**

The online version contains supplementary material available at 10.1186/s40337-021-00439-z.

## Main text

Anorexia nervosa (AN) is a severe and complex illness with high mortality, poorly understood pathophysiology and lack of efficacious treatment. There is a pressing need for interventions to modify causal and maintaining factors. The effects of nutrition on the brain and behaviour in AN is of particular interest, though an area of limited research. One area of consideration is the brain noradrenergic system and the role of tyrosine as an adjunct treatment [1]. This hypothesis relies on increasing blood tyrosine sufficiently to facilitate brain catecholamine synthesis. There is some evidence suggesting blood tyrosine may be lowered in AN [2]. In healthy adults, peak tyrosine occurs approximately two to three hours post-supplementation and approaches baseline by eight hours [3, 4]. No studies could be found reporting pharmacological response or safety of tyrosine supplementation in adolescents with AN. We aimed to explore the pharmacokinetics of tyrosine loading in adolescents with anorexia nervosa and healthy peers.

We studied the response to a single 2.5 g oral L-tyrosine load (capsules) in two female adolescents with AN and two healthy peers, while on a low protein, low biogenic amine diet (day prior and initial day of testing, fasting eight hours before initial bloods). Participants with AN then continued 2.5 g tyrosine twice daily for 12 weeks. Peer recruitment was via volunteer posters in health settings (October to December 2006) and by staff approaching patients with AN admitted to a tertiary hospital in New South Wales, Australia (February 2007 to March 2010). Exclusion criteria included use of amino acid supplements within three months, medical instability, severe medical or neurological illness, phenylketonuria, drug or alcohol abuse within six months or requiring noradrenergic, combined noradrenergic or stimulant medication. Diagnosis was confirmed by the Eating Disorders Examination interview (child version) [5]. Food diaries were maintained and meals for those with AN supervised by nursing staff. Participants were reviewed by the pediatrician four hours after initial supplementation and monitored by nursing staff for eight hours. Participants with AN were monitored by nursing and medical staff for the first four days.

Blood tyrosine level was the main outcome measure and initially taken four hours before (fasting), immediately before supplementation and at one, two, three, four, six and eight hours post-supplementation. For the second stage, blood was taken at baseline and two hours post-supplementation at weeks one, six and 12 in participants with AN. For healthy peers, heparinized plasma were analyzed by high performance liquid chromatography with electrochemical detection. Due to laboratory resource issues for those with AN, electrospray tandem mass spectrometry in dried-blood-spots with underivatised samples was used. Tyrosine levels in blood spots and plasma from the same samples correlated well within the laboratory previously, suggesting limited variation would occur between plasma and blood spot. 24-h dietary recalls were collected at four time points in AN to coincide with bloods. Psychological and neurocognitive measures were completed. See Additional File 1 for details of testing timepoints.

**Fig. 1:**
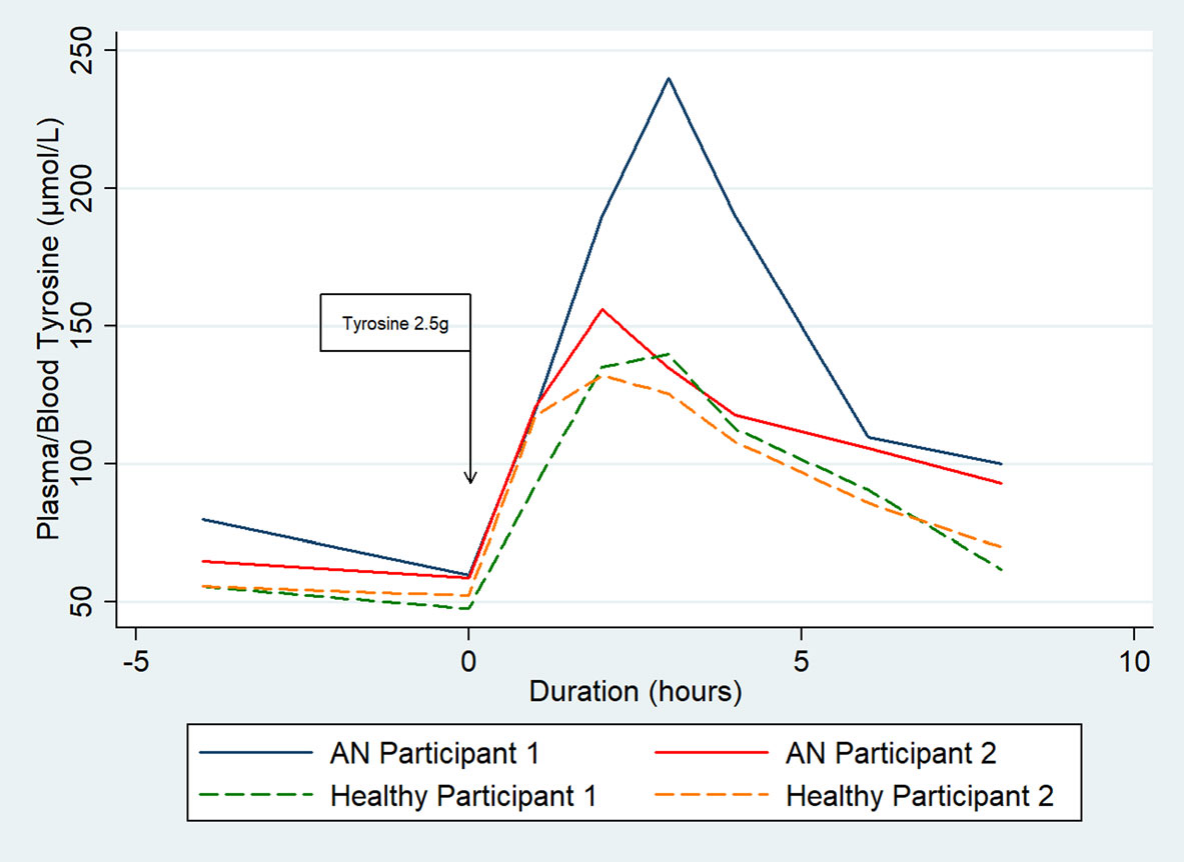
Blood Tyrosine in Anorexia Nervosa (*n* = 2) and Control Plasma Tyrosine (*n* = 2) Response to Tyrosine Load

Additional File 1 provides demographic, dosage and dietary intake information. On day one, baseline tyrosine concentrations were similar for all four participants (48-60 μmol/L) (Fig. 1). Peak tyrosine was observed at approximately two to three hours (132-240 μmol/L) and approached baseline by eight hours (62-100 μmol/L). Percentage change in tyrosine (between trough and peak) was 152–194% in healthy peers. Participant 2 with AN had a similar tyrosine percentage change to healthy peers (164%). Participant 1 with AN had a notably higher peak tyrosine response (300% change). There was a sustained rise in blood tyrosine over 12 weeks in Participant 1 which diminished over time (Additional File 1). In Participant 2, morning trough and peak levels normalized over time.

Over the study, percent expected body weight (actual BMI by 50th Centile BMI on growth charts) [6] remained essentially unchanged (80%) in Participant 1, while Participant 2 was relatively weight-restored (96%). No side effects were observed, measured or reported by participants or staff. Participant 1 was admitted to a mental health ward ten-and-a-half weeks after commencing supplementation. No decline was measured in psychological tests (Additional File 1), although some increase was evident in Total Difficulties.

This study contributes to the limited knowledge around the effects of L-tyrosine in AN by exploring the pharmacokinetics of tyrosine loading. Peak tyrosine occurred two to three hours post-supplementation and approached baseline by eight hours [3]. Participants with AN and healthy peers exceeded the 30–50% increase suggestive of facilitating brain tyrosine changes in rats [7, 8]. Lowered blood tyrosine in AN was not observed in baseline results, perhaps due to active re-feeding. Variations in blood tyrosine response requires further exploration. Factors such as age, gender, tyrosine dosage, the exogenous effects of food, nutritional status, re-feeding, medications, vomiting, biological adaption to tyrosine over time and metabolic variation could all contribute. Further exploration of potential effects of continued tyrosine administration is required. A controlled trial may provide further information on whether any of the observed effects are generalisable.

## Supplementary Information

**Additional file 1.** Contains additional results for readers wishing to read further. This includes study participants, supplemental dosage, dietary intakes, blood tyrosine values, percent expected body weight and results of psychological tests.

## Data Availability

The datasets generated and/or analysed during the current study are not publicly available due to the small sample size and participant privacy though are available from the corresponding author on reasonable request.

## Abbreviation

AN: anorexia nervosa
