# Supplementary material for: Progressing our understanding of the impacts of nutrition on the brain and behaviour in anorexia nervosa: a tyrosine case study example
Source: J Eat Disord. 2021 Jul 13;9:86. doi: 10.1186/s40337-021-00439-z (PMC8278653; doi:10.1186/s40337-021-00439-z)
Supplement: Supplementary file 1 — Additional file 1. Contains additional results for readers wishing to read further. This includes study participants, supplemental dosage, dietary intakes, blood tyrosine values, percent expected body weight and results of psychological tests. [file 40337_2021_439_MOESM1_ESM.docx]

**Progressing our understanding of the impacts of nutrition on the brain and behaviour in anorexia nervosa: a tyrosine case study example – Additional File 1**

Intervention: tyrosine 5g per day for 12 weeks

Baseline Testing:

Eating Disorders Examination (Child)

Cognitive Function Tests

Anxiety Inventory

Depression Inventory

Obsessive Compulsive Inventory

Strengths and Difficulties Questionnaire

24 hour recall

Height

Weight

Blood

Urine

Week 1 Testing:

Anxiety Inventory

24 hour recall

Blood

Urine

Week 6 Testing:

Anxiety Inventory

Depression Inventory

Obsessive Compulsive Inventory

Strengths and Difficulties Questionnaire

24 hour recall

Height

Weight

Blood

Urine

Week 12 Testing:

Eating Disorders Examination (Child)

Cognitive Function Tests

Anxiety Inventory

Depression Inventory

Obsessive Compulsive Inventory

Strengths and Difficulties Questionnaire

24 hour recall

Height

Weight

Blood

Urine

Questionnaire

**Diagram: Testing Schedule for Participants with Anorexia Nervosa During 12 weeks of Tyrosine Supplementation**

**Study Participants:**

Healthy peers were both female, aged 14 years and had a percentage expected body weight of 100-110%. Neither scored in the clinical range for all psychological screening tests. Participant 1 with AN was 15 years of age, weighed 46kg at baseline and had been amenorrheic for three months. Participant 2 was 12 years of age, weighed 37kg at baseline and remained pre-menarchal. Both AN participants had a relatively short duration of illness (three months) and similar expected body weights at baseline (80-82%). Neither participant with AN had received treatment prior to the recent hospital admission and had no comorbid medical or psychiatric diagnoses. Both participants with AN were taking multivitamin, thiamine and phosphate supplements at baseline, though no other medications. Both participants were refed on the pediatric ward for the initial 2 weeks of the study, then attended CAMHS for weekly family therapy interventions. Participant 1 with AN had a secondary diagnosis of obsessive compulsive disorder at completion of the study. Both participants had consumed Olanzapine (an antipsychotic) at times during the study and Participant 1 also consumed Lorazepam (a benzodiazepine) towards the end of the study. Participant 1 reported several self-induced vomiting episodes before and during the study, though denied other purgative behavior. Binge eating and excessive exercise behavior were denied by both participants.

Table 1: Estimated Supplement Dosage and Dietary Macronutrient and LNAA Intakes Over Twelve Weeks (*n*=2)

| **Item** | **Participant 1**  **Mean, *n*=1** | **Participant 2**  **Mean, *n*=1** |
| --- | --- | --- |
|  |  |  |
| Supplement Dosage (mg/kg) | 5g/day (109.7mg/kg) | 5g/day (121.4mg/kg) |
| Unused Supplement Count (per 500mg capsules) | 3 (<1%) | 23 (3%) |
| Energy (kJ/day) | 7065.7 | 8752.4 |
| Fat (g/day) | 53.1 | 89.9 |
| Protein (g/day) | 54.4 | 93.8 |
| Carbohydrate (g/day) | 204.2 | 265.9 |
| Fibre (g/day) | 13.7 | 20.3 |
| Fluid (g/day) | 2122.4 | 2235.7 |
| Tyrosine (g/day) | 2.2 | 3.7 |
| Phenylalanine (g/day) | 2.4 | 4.1 |
| Tryptophan (g/day) | 0.7 | 1.0 |
| Leucine (g/day) | 4.5 | 7.2 |
| Isoleucine (g/day) | 2.6 | 4.2 |
| Valine (g/day) | 3.1 | 5.0 |
| Methionine (g/day) | 1.2 | 2.2 |
|  |  |  |

NB: LNAA denotes large neutral amino acid

**Table 2. Blood Tyrosine Response to Tyrosine Load in AN Over Twelve weeks (*n*=2) (µmol/L)**

| **Time Point**  **(µmol/L)** | **Time 1**  **(0 Hours, Estimated Trough)** | **Time 2**  **(2 Hours, Estimated Peak)** | **Absolute Difference**  **(% Change)** |
| --- | --- | --- | --- |
|  |  |  |  |
| Baseline |  |  |  |
| Participant 1 | 60 | 190† | 130 (217%) |
| Participant 2 | 59 | 156† | 97 (164%) |
| Week 1 |  |  |  |
| Participant 1 | 140† | 390† | 250 (179%) |
| Participant 2 | 125† | 372† | 247 (198%) |
| Week 6 |  |  |  |
| Participant 1 | 140† | 290† | 150 (107%) |
| Participant 2 | 91 | 278† | 187 (205%) |
| Week 12 |  |  |  |
| Participant 1 | 230† | 340† | 110 (48%) |
| Participant 2 | 50 | 161† | 111 (222%) |
|  |  |  |  |

Note: Time 1 denotes the time of supplement administration and Time 2 denotes two hours post-tyrosine administration. † denotes outside the reference range.

**Table 3: Participant Change in Percent Expected Body Weight Over Time (*n*=2)**

|  |  |  |  |
| --- | --- | --- | --- |
| **Participant** | **Baseline** | **Week 6**  **(% Change)** | **Week 12**  **(% Change)** |
|  |  |  |  |
| Participant 1 | 82% | 84% (2%) | 80% (-2%) |
| Participant 2 | 80% | 92% (12%) | 96% (16%) |
|  |  |  |  |

Note: %EBW denotes percentage expected body weight; and % Change

denotes percentage change from baseline in study participants.

**Table 4. Change in Participant Eating Disorders Psychopathology Following Twelve Weeks of Tyrosine Supplementation (*n*=2)**

| **Eating Disorders Examination**  **(Child Version) Scales** | **Baseline** | **Week 12** | **Absolute Difference**  **(% Change)** | **RCI** |
| --- | --- | --- | --- | --- |
|  |  |  |  |  |
| **Participant 1** |  |  |  |  |
| Restraint | 6.0† | 6.0† | 0 (0%) | 0.00 |
| Eating Concern | 3.4† | 4.6† | 1.2 (35%) | 1.63 |
| Weight Concern | 5.2† | 4.6† | -0.6 (-12%) | -0.76 |
| Shape Concern | 4.9† | 3.8† | -1.13 (-23%) | -1.39 |
| Global Score | 4.9† | 4.7† | -0.13 (-3%) | -0.37 |
| **Participant 2** |  |  |  |  |
| Restraint | 5.0† | 2.2† | -2.8 (-56%) | -2.50* |
| Eating Concern | 3.4† | 4.2† | 0.8 (24%) | 1.09 |
| Weight Concern | 5.2† | 4.6† | -0.6 (-12%) | -0.76 |
| Shape Concern | 5.9† | 4.9† | -1.0 (-17%) | -1.26 |
| Global Score | 4.9† | 4.0† | -0.9 (-18%) | -1.65* |
|  |  |  |  |  |

Note: Measure Eating Disorders Examination [[1](#_ENREF_1)]; Absolute Difference denotes the difference in participant baseline and follow up scores; % Change denotes percentage change in participant raw scores over time; RCI denotes Reliable Change Index [[2](#_ENREF_2)]; † denotes within the clinically significant range, based on a z score of two or more (calculated from Wade et al, 2008) [[3](#_ENREF_3)]; and * denotes a significant reliable change over time [[4](#_ENREF_4)].

**Table 5: Change in Participant Anxiety Following Twelve Weeks of Tyrosine Administration (*n*=2)**

|  | **Baseline** | **Week 1** | | **Week 6** | | **Week 12** | | |  |
| --- | --- | --- | --- | --- | --- | --- | --- | --- | --- |
| **Anxiety Scale** | **Raw Score**  **(Std Score)**  **[Percentile]** | **Raw Score**  **(Std Score)**  **[Percentile]** | **Absolute Difference (% Change)** | **Raw Score**  **(Std Score)**  **[Percentile]** | **Absolute Difference (% Change)** | | **Raw Score**  **(Std Score)**  **[Percentile]** | **Absolute Difference (% Change)** | |
|  |  |  |  |  |  | |  |  | |
| **Participant 1** |  |  |  |  |  | |  |  | |
| State Anxiety | 59 (64) [92] | 57 (63) [89] | -2 (-3%) | 42 (51) [58] | -17 (-29%) | | 51 (58) [82] | -8 (-14%) | |
| Trait Anxiety | 66 (74) [98] † | 50 (59) [80] ‡ | -16 (-24%) | 59 (67) [95] | -7 (-11%) | | 57 (65) [89] | -9 (-14%) | |
| **Participant 2** |  |  |  |  |  | |  |  | |
| State Anxiety | 53 (60) [86] | 57 (63) [89] | 4 (8%) | 58 (64) [90] | 5 (9%) | | 52 (59) [84] | -1 (-2%) | |
| Trait Anxiety | 71 (78) [99] † | 65 (73) [98] † | -6 (-8%) | 55 (63) [91] ‡ | -16 (-23%) | | 48 (57) [76] | -23 (-32%) | |
|  |  |  |  |  |  | |  |  | |

Note: Measure - State Trait Anxiety Inventory Form Y-1 [[5](#_ENREF_5)]; Std Score denotes standard score; % Change denotes percentage change in raw score over time; † denotes clinically significant anxiety symptoms based on 2 or more standard deviations from the mean (T score of 70 or more) [[6](#_ENREF_6)]; and ‡ denotes a clinically significant change (moved into or out of the clinically significant range).

**Table 6: Change in Participant Depressive Symptomatology Over Twelve Weeks of Tyrosine Administration (*n*=2)**

|  | **Baseline** | | **Week 6** | | | | | **Week 12** | | | | | | | |  |  |
| --- | --- | --- | --- | --- | --- | --- | --- | --- | --- | --- | --- | --- | --- | --- | --- | --- | --- |
| **Symptom Scale** | **Raw Score**  **(T Score)** | **Interpretive Guideline** | | **Raw Score**  **(T Score)** | **Interpretive Guideline** | | | | **Absolute Difference**  **(% Change)** | **Raw Score**  **(T Score)** | | **Interpretive Guideline** | | | **Absolute Difference**  **(% Change)** | |  |
|  |  |  | |  |  | | | |  |  | |  | | |  | |  |
| Negative Mood |  |  | |  |  | | | |  |  | |  | | |  | |  |
| Participant 1 | 7 (75) † | Very Much Above Average | | 5 (64) ‡ | Above Average | | | | -2 (-29%) | 6 (70) †‡ | | Very Much Above Average | | | -1 (-14%) | |  |
| Participant 2 | 8 (81) † | Very Much Above Average | | 9 (86) † | Very Much Above Average | | | | 1 (13%) | 3 (54) ‡ | | Average | | | -5 (-63%) | |  |
| Interpersonal Problems |  |  | |  |  | | | |  |  | |  | | |  | |  |
| Participant 1 | 2 (64) | Above Average | | 3 (74) †‡ | Very Much Above Average | | | | 1 (50%) | 4 (84) † | | Very Much Above Average | | | 2 (100%) | |  |
| Participant 2 | 1 (54) | Average | | 0 (43) | Slightly Below Average | | | | -1 (-100%) | 1 (54) | | Average | | | 0 (0%) | |  |
| Ineffectiveness |  |  | |  |  | | | |  |  | |  | | |  | |  |
| Participant 1 | 2 (52) | Average | | 2 (52) | Average | | | | 0 (0%) | 4 (66) †‡ | | Very Much Above Average | | | 2 (100%) | |  |
| Participant 2 | 5 (74) † | Very Much Above Average | | 5 (74) † | Very Much Above Average | | | | 0 (0%) | 2 (52) ‡ | | Average | | | -3 (-60%) | |  |
| Anhedonia |  |  | |  |  | | | |  |  | |  | | |  | |  |
| Participant 1 | 12 (82) † | Very Much Above Average | | 7 (63) ‡ | Above Average | | | | -5 (-42%) | 7 (63) | | Above Average | | | -5 (-42%) | |  |
| Participant 2 | 12 (82) † | Very Much Above Average | | 5 (56) ‡ | Slightly Above Average | | | | -7 (-58%) | 3 (49) | | Average | | | -9 (-75%) | |  |
| Negative  Self-Esteem |  |  | |  |  | | | |  |  | |  | | |  | |  |
| Participant 1 | 4 (64) | Above Average | | 4 (64) | Above Average | | | | 0 (0%) | 4 (64) | | Above Average | | | 0 (0%) | |  |
| Participant 2 | 5 (70) † | Very Much Above Average | | 4 (64) ‡ | Above Average | | | | -1 (-20%) | 3 (58) | | Slightly Above Average | | | -2 (-40%) | |  |
| Total Score |  |  | |  |  | | | |  |  | |  | | |  | |  |
| Participant 1 | 27 (78) † | Very Much Above Average | | 21 (68) † | Very Much Above Average | | | | -6 (-22%) | 25 (74) † | | Very Much Above Average | | | -2 (-7%) | |  |
| Participant 2 | 31 (84) † | Very Much Above Average | | 23 (71) † | Very Much Above Average | | | | -8 (-26%) | 12 (54) ‡ | | Average | | | -19 (-61%) | |  |
|  |  |  | |  | |  |  | | | |  | |  |  | | | |

Note: Measure – Children’s Depression Inventory [[7](#_ENREF_7)]; Absolute Difference denotes the difference in participant baseline and follow-up raw scores; % Change denotes percentage change in raw score from baseline; † denotes clinically significant depressive symptomatology, based on guidelines from Kovacs (1992); and ‡ denotes a clinically significant change (moved into or out of the clinically significant range).

**Table 7: Change in Participant Obsessive Compulsive Symptomatology Following Twelve Weeks of Tyrosine Supplementation (*n*=2)**

| **Symptom Scale** | **Baseline** | **Week 6** | **Absolute Change**  **(% Change)** | **Week 12** | **Absolute Change**  **(% Change)** |
| --- | --- | --- | --- | --- | --- |
|  |  |  |  |  |  |
| Compulsions Symptoms |  |  |  |  |  |
| Participant 1 | 33 | 33 | 0 (0%) | 28 | -5 (-15%) |
| Participant 2 | 33 | 26 | -7 (-21%) | 23 | -10 (-30%) |
| Compulsions Severity |  |  |  |  |  |
| Participant 1 | 21 | 18 | -3 (-14%) | 12 | -9 (-43%) |
| Participant 2 | 17 | 5 | -12 (-71%) | 14 | -3 (-18%) |
| Obsessions Symptoms |  |  |  |  |  |
| Participant 1 | 24 | 18 | -6 (-25%) | 18 | -6 (-25%) |
| Participant 2 | 23 | 17 | -6 (-26%) | 17 | -6 (-26%) |
| Obsessions Severity |  |  |  |  |  |
| Participant 1 | 7 | 16 | 9 (129%) | 13 | 6 (86%) |
| Participant 2 | 16 | 9 | -7 (-44%) | 14 | -2 (-13%) |
| Total Impairment |  |  |  |  |  |
| Participant 1 | 28† | 34† | 6 (21%) | 25† | -3 (-11%) |
| Participant 2 | 33† | 14 ‡ | -19 (-58%) | 28†‡ | -5 (-15%) |
|  |  |  |  |  |  |

Note: Measure – Children’s Obsessive Compulsive Inventory [[8](#_ENREF_8)]; Absolute Difference denotes the difference in baseline and follow-up raw scores; % Change denotes percentage change in raw score from baseline; † denotes clinically significant obsessive compulsive total impairment symptoms, based on a score of ≥17 [[8](#_ENREF_8)]; and ‡ denotes a clinically significant change (moved into or out of the clinically significant range).

**Table 8: Strengths and Difficulties Questionnaire Results for Study Participants (*n*=2)**

| **Item** | **Baseline** | | **Week 6** | | | **Week 12** | | |
| --- | --- | --- | --- | --- | --- | --- | --- | --- |
|  | **Raw Score** | **Classification** | **Raw Score** | **Classification** | **Absolute Difference**  **(% Change)** | **Raw Score** | **Classification** | **Absolute Difference**  **(% Change)** |
|  |  |  |  |  |  |  |  |  |
| **Emotional Problems** |  |  |  |  |  |  |  |  |
| Patient |  |  |  |  |  |  |  |  |
| Participant 1 | 4 | Normal | 7†‡ | Abnormal | 3 (75%) | 7† | Abnormal | 3 (75%) |
| Participant 2 | 9† | Abnormal | 9† | Abnormal | 0 (0%) | 5‡ | Normal | -4 (-44%) |
| Parent |  |  |  |  |  |  |  |  |
| Participant 1 | 1 | Normal | . | . | N/A | 6†‡ | Abnormal | 5 (500%) |
| Participant 2 | 4 | Borderline | 5†‡ | Abnormal | 1 (25%) | 8† | Abnormal | 4 (100%) |
| **Conduct Problems** |  |  |  |  |  |  |  |  |
| Patient |  |  |  |  |  |  |  |  |
| Participant 1 | 6† | Abnormal | 4‡ | Borderline | -2 (-33%) | 5†‡ | Abnormal | -1 (-17%) |
| Participant 2 | 1 | Normal | 1 | Normal | 0 (0%) | 2 | Normal | 1 (100%) |
| Parent |  |  |  |  |  |  |  |  |
| Participant 1 | 4† | Abnormal | . | . | N/A | 5† | Abnormal | 1 (25%) |
| Participant 2 | 2 | Normal | 3 | Borderline | 1 (50%) | 2 | Normal | 0 (0%) |
| **Hyperactivity** |  |  |  |  |  |  |  |  |
| Patient |  |  |  |  |  |  |  |  |
| Participant 1 | 3 | Normal | 8†‡ | Abnormal | 5 (167%) | 5‡ | Normal | 2 (67%) |
| Participant 2 | 8† | Abnormal | 8† | Abnormal | 0 (0%) | 5‡ | Normal | -3 (-38%) |
| Parent |  |  |  |  |  |  |  |  |
| Participant 1 | 6 | Borderline | . | . | N/A | 5 | Normal | -1 (-17%) |
| Participant 2 | 0 | Normal | 3 | Normal | 3 (N/A) | 4 | Normal | 4 (N/A) |
| **Peer Problems** |  |  |  |  |  |  |  |  |
| Patient |  |  |  |  |  |  |  |  |
| Participant 1 | 4 | Borderline | 6†‡ | Abnormal | 2 (50%) | 6† | Abnormal | 2 (50%) |
| Participant 2 | 1 | Normal | 3 | Normal | 2 (200%) | 3 | Normal | 2 (200%) |
| Parent |  |  |  |  |  |  |  |  |
| Participant 1 | 0 | Normal | . | . | N/A | 4†‡ | Abnormal | 4 (N/A) |
| Participant 2 | 0 | Normal | 0 | Normal | 0 (0%) | 0 | Normal | 0 (N/A) |
| **Item** | **Baseline** | | **Week 6** | | | **Week 12** | | |
|  | **Raw Score** | **Classification** | **Raw Score** | **Classification** | **Absolute Difference**  **(% Change)** | **Raw Score** | **Classification** | **Absolute Difference**  **(% Change)** |
| **Prosocial Behaviour** |  |  |  |  |  |  |  |  |
| Patient |  |  |  |  |  |  |  |  |
| Participant 1 | 8 | Normal | 9 | Normal | 1 (13%) | 6 | Normal | -2 (-25%) |
| Participant 2 | 10 | Normal | 10 | Normal | 0 (0%) | 6 | Normal | -4 (-40%) |
| Parent |  |  |  |  |  |  |  |  |
| Participant 1 | 8 | Normal | . | . | N/A | 6 | Normal | -2 (-25%) |
| Participant 2 | 8 | Normal | 7 | Normal | -1 (-13%) | 7 | Normal | -1 (-13%) |
| **Total Difficulties** |  |  |  |  |  |  |  |  |
| Patient |  |  |  |  |  |  |  |  |
| Participant 1 | 17 | Borderline | 25†‡ | Abnormal | 8 (47%) | 23† | Abnormal | 6 (35%) |
| Participant 2 | 19 | Borderline | 21†‡ | Abnormal | 2 (11%) | 15‡ | Normal | -4 (-21%) |
| Parent |  |  |  |  |  |  |  |  |
| Participant 1 | 11 | Normal | . | . | N/A | 20†‡ | Abnormal | 9 (82%) |
| Participant 2 | 6 | Normal | 11 | Normal | 5 (83%) | 14 | Borderline | 8 (133%) |
| **Total Impact** |  |  |  |  |  |  |  |  |
| Patient |  |  |  |  |  |  |  |  |
| Participant 1 | 4† | Abnormal | 5† | Abnormal | 1 (25%) | 6† | Abnormal | 2 (50%) |
| Participant 2 | 9† | Abnormal | 0‡ | Normal | -9 (-100%) | 3†‡ | Abnormal | -6 (-67%) |
| Parent |  |  |  |  |  |  |  |  |
| Participant 1 | 0 | Normal | . | . | N/A | 8†‡ | Abnormal | 8 (N/A) |
| Participant 2 | 10† | Abnormal | 7† | Abnormal | -3 (-30%) | 7† | Abnormal | -3 (-30%) |
|  |  |  |  |  |  |  |  |  |

Note: Measure – Strenths and Difficulties Questionnaire [[9](#_ENREF_9)]; Absolute Difference denotes the difference in participant baseline and follow-up raw scores; % Change denotes percentage change in raw score from baseline; “.” Denotes missing data; † denotes clinically significant symptoms based on scores fitting within the “abnormal” classification according to test instructions [[9](#_ENREF_9), [10](#_ENREF_10)]; and ‡ denotes a clinically significant change (moved into or out of the clinically significant range).

**Table 9: Participant Change in Neurocognitive Performance Following Twelve Weeks of Supplementation (n=2)**

| **Cognitive**  **Function Test** | **Baseline Raw Score (Converted Score)** | **Ability Range** | **Week 12 Raw Score (Converted Score)** | **Ability Range** | **Absolute Difference**  **(% Change)** | **RCI_PE_ (RCI_EstSRB_)** |
| --- | --- | --- | --- | --- | --- | --- |
|  |  |  |  |  |  |  |
| **Reading** **Test** [Standard Score] |  |  |  |  |  |  |
| Participant 1 | 50 (118) | High Average | 48 (113) | High Average | -2 (-4%) | -0.48 (-0.30) |
| Participant 2 | 46 (113) | High Average | 45 (110) | High Average | -1 (-2%) | -0.31 (-0.12) |
| **Rey Complex Figure Test** |  |  |  |  |  |  |
| Copy Accuracy [Percentile] |  |  |  |  |  |  |
| Participant 1 | 32 (>16) | Average | 31 (11-16) | Low Average | -1 (-2%) | N/A |
| Participant 2 | 32 (>16) | Average | 35 (>16) | Average | 3 (9%) | N/A |
| Delayed Recall [T score] |  |  |  |  |  |  |
| Participant 1 | 24 (51) | Average | 18 (39) | Low Average | -6 (-27%) | N/A |
| Participant 2 | . | N/A | 20 (46) | Average | . | N/A |
| Central Coherence Index (Copy) |  |  |  |  |  |  |
| Participant 1 | 1.76 | N/A | 1.38 | N/A | -0.38 (-22) | N/A |
| Participant 2 | 0.94 | N/A | 1.67 | N/A | 0.73 (78%) | N/A |
| Central Coherence Index (Delayed) |  |  |  |  |  |  |
| Participant 1 | 1.18 | N/A | 1.48 | N/A | 0.30 (25%) | N/A |
| Participant 2 | . | N/A | 1.92 | N/A | . |  |
| **Verbal Fluency Test** [Scaled Score] |  |  |  |  |  |  |
| Participant 1 | 26 (8) | Average | 37 (12) | High Average | 11 (42%) | N/A |
| Participant 2 | 33 (12) | Average | 32 (12) | Average | -1 (-3%) | N/A |
| **Tower of London** [z Score] |  |  |  |  |  |  |
| Participant 1 | 28 (-2.4) † | WBE | 35 (1.3) ‡ | High Average | 7 (25%) | N/A |
| Participant 2 | 26 (-2.4) † | WBE | 34 (1.0) ‡ | High Average | 8 (31%) | N/A |
| **Stroop Task** [T score] |  |  |  |  |  |  |
| Stroop Word |  |  |  |  |  |  |
| Participant 1 | 97 (48) | Average | 111 (54) | Average | 14 (10%) | N/A |
| Participant 2 | 95 (50) | Average | 93 (50) | Average | -2 (-2%) | N/A |
| Stroop Colour |  |  |  |  |  |  |
| Participant 1 | 76 (48) | Average | 75 (48) | Average | -1 (-1%) | N/A |
| Participant 2 | 70 (50) | Average | 63 (44) | Average | -7 (-10%) | N/A |
| Stroop Colour Word |  |  |  |  |  |  |
| Participant 1 | 41 (48) | Average | 60 (66) | Superior | 19 (46%) | N/A |
| Participant 2 | 40 (52) | Average | 30 (42) | Low Average | -10 (-25%) | N/A |
| **Cognitive**  **Function Test** | **Baseline Raw Score (Converted Score)** | **Ability Range** | **Week 12 Raw Score (Converted Score)** | **Ability Range** | **Absolute Difference**  **(% Change)** | **RCI_PE_ (RCI_EstSRB_)** |
|  |  |  |  |  |  |  |
| **Verbal Paired Associate [N/A]** |  |  |  |  |  |  |
| Learning Trials |  |  |  |  |  |  |
| Participant 1 | 21 (N/A) | N/A | 19 (N/A) | N/A | -2 (-10%) | N/A |
| Participant 2 | 23 (N/A) | N/A | 23 (N/A) | N/A | 0 (0%) | N/A |
| Delayed |  |  |  |  |  |  |
| Participant 1 | 8 (N/A) | N/A | 7 (N/A) | N/A | -1 (-13%) | N/A |
| Participant 2 | 8 (N/A) | N/A | 8 (N/A) | N/A | 0 (0%) | N/A |
| **Digit-Symbol** [Scaled Score] |  |  |  |  |  |  |
| Participant 1 | 73 (13) | High Average | 74 (13) | High Average | 1 (1%) | -0.65 (-0.65) |
| Participant 2 | 81 (17) † | Very Superior | 70 (14) | Superior ‡ | -11 (-14%) | -2.43*(-2.43)* |
| **Visual Learning** |  |  |  |  |  |  |
| Learning Trials [Scaled Score] |  |  |  |  |  |  |
| Participant 1 | 34 (11) | Average | 38 (13) | High Average | 4 (12%) | N/A |
| Participant 2 | 24 (8) | Average | 26 (9) | Average | 2 (8%) | N/A |
| Delayed [N/A] |  |  |  |  |  |  |
| Participant 1 | 7 (N/A) | N/A | 9 (N/A) | N/A | 2 (29%) | N/A |
| Participant 2 | 5 (N/A) | N/A | 7 (N/A) | N/A | 2 (40%) | N/A |
| **Matching** [Standard Score] |  |  |  |  |  |  |
| Participant 1 | 32 (87) | Low Average | 30 (80) | Low Average | -2 (-6%) | -0.56 (-0.40) |
| Participant 2 | 41 (119) | High Average | 41 (119) | High Average | 0 (0%) | -0.18 (-0.34) |
| **Trail Making A** (z Score) |  |  |  |  |  |  |
| Participant 1 | 24 (-1.4) | Borderline | 10 (0.9) | High Average | -14 (-58%) | N/A |
| Participant 2 | 21 (-0.7) | Low Average | 10 (0.7) | Average | -11 (-52%) | N/A |
| **Trail Making B** (z Score) |  |  |  |  |  |  |
| Participant 1 | 95 (-5.3) † | WBE | 32 (-0.4) ‡ | Average | -63 (-66%) | N/A |
| Participant 2 | 25 (0.3) | Average | 19 (0.9) | High Average | -6 (-24%) | N/A |
| **Design Fluency** (T score) |  |  |  |  |  |  |
| Participant 1 | . | N/A | 117 (54) | [Average] | . | N/A |
| Participant 2 | 89 (42) | Low Average | . | N/A | . | N/A |
|  |  |  |  |  |  |  |

Note: Absolute Difference denotes the difference in baseline and follow-up raw scores; % Change denotes the percentage change in raw scores over time; RCI_PE_ denotes reliable change index plus practice effects formula (Chelune et al, 1993) [[11](#_ENREF_11)]; RCI_EstSRB_ denotes reliable change index estimated standardised regression-based formula (Maassen et al, 2006) [[12](#_ENREF_12)]; “.” denotes missing data; † denotes within the clinically significant range (2 SD from the mean or equivalent) [[6](#_ENREF_6)]; ‡ denotes a clinically significant change (moved into or out of the clinically significant range); * denotes a significant reliable change over time [[4](#_ENREF_4)]; and WBE denotes Well Below Expected.

A standardised battery of cognitive function tests were administered by experienced clinical psychologists at baseline and week 12. These were: Rey Complex Figure Test (Meyer and Meyer) initially copy and 30 minute recall [[13](#_ENREF_13)], Verbal Fluency (FAS) Condition One (Baron) [[14](#_ENREF_14)], Tower Task (Krikorian) [[15](#_ENREF_15)], Stroop Color-Word task (Golden) [[16](#_ENREF_16)], Verbal Paired Associate Learning (Wechsler Memory Scale, Revised) [[17](#_ENREF_17)], Digit Symbol-Coding (Wechsler Intelligence Scale for Children, Fourth Edition) [[18](#_ENREF_18)], Visual Learning (Wide Range Assessment of Memory and Learning) [[19](#_ENREF_19)], Matching (Wide Range Assessment of Visual-Motor Abilities) [[20](#_ENREF_20)], Trail Making (Reitan) [[21](#_ENREF_21)] and Design Fluency [[22](#_ENREF_22)]. The Wide Range Achievement Test-3 Reading Test [[23](#_ENREF_23)] was used as a measure of executive function. An experienced neuropsychologist converted participant test scores to normative data and applied ability ranges. In order to minimise systematic error (e.g. practice effects) or measurement error (e.g. test unreliability) in psychological tests, reliable change index (RCI) methods were used to examine change in psychological tests [[24](#_ENREF_24)].

1. Watkins, B., et al., Reliability and Validity of the Child Version of the Eating Disorders Examination : A Preliminary Investigation. International Journal of Eating Disorders, 2005. 38: p. 183-187.

2. Jacobson, N.S. and P. Truax, Clinical Significance: A Statistical Approach to Defining Meaningful Change in Psychotherapy Research. Journal of Consulting and Clinical Psychology, 1991. 59(1): p. 12-19.

3. Wade, T., S. Byrne, and R. Bryant-Waugh, The Eating Disorder Examination: Norms and Construct Validity with Young and Middle Adolescent Girls. International Journal of Eating Disorders, 2008. 41: p. 551–558.

4. Duff, K., Evidence-Based Indicators of Neuropsychological Change in the Individual Patient: Relevant Concepts and Methods. Archives of Clinical Neuropsychology, 2012. 27: p. 248–261.

5. Spielberger, C.D., et al., Measuring Anxiety and Anger in the State-Trait Anxiety Inventory (STAI) and the State-Trait Anger Expression Inventory (STAXI), in The Use of Psychological Testing for Treatment Planning and Outcome Assessment. 1999, Lawrence Erlbaum Associates: Mahwah.

6. Gregory, R.J., Norms and Reliability, in Psychological Testing, History, Principles, and Applications, Sixth Edition. 2011, Allyn and Bacon: Boston. p. 67-86.

7. Kovacs, M., Children’s Depression Inventory Manual. 1992, New York: MultiHealth Systems Inc.

8. Shafran, R., et al., The Preliminary Development of a New Self-Report Measure for OCD in Young People. Journal of Adolescence, 2003. 26: p. 137-142.

9. Goodman, R., The Extended Version of the Strengths and Difficulties Questionnaire as a Guide to Child Psychiatric Caseness and Consequent Burden. Journal of Child Psychology and Psychiatry, 1999. 40(5): p. 791-799.

10. Goodman, R., H. Meltzer, and V. Bailey, The Strengths and Difficulties Questionnaire: A Pilot Study on the Validity of the Self-Report Version. European Child & Adolescent Psychiatry, 1998. 7: p. 125-130.

11. Chelune, G.J., et al., Individual Change After Epilepsy Surgery: Practice Effects and Base-Rate Information. Neuropsychology, 1993. 7(1): p. 41-52.

12. Maassen, G.H., E.R. Bossema, and N. Brand, Reliable Change Assessment with Practice Effects in Sport Concussion Research: a Comment on Hinton-Bayre. British Journal of Sports Medicine, 2006. 40(10): p. 829–833.

13. Meyers, J.E. and K.R. Meyers, Rey Complex Figure Test and Recognition Trial, Professional Manual. 1995, USA: Psychological Assessment Resources Incorporated.

14. Baron, I.S., Neuropsychological Evaluation of the Child. 2004, New York: Oxford University Press.

15. Krikorian, R., J. Bartok, and N. Gay, Tower of London Procedure – A Standard Method and Developmental Data. Journal of Clinical and Experimental Neuropsychology, 1994. 16(6): p. 840-50.

16. Golden, C.J., The Stroop Color and Word Test: A Manual for Clinical and Experimental Uses. 1978, Chicago: Stoelting.

17. Wechsler, D., Wechsler Memory Scale – Revised Manual. 1987, San Antonio: The Psychological Corporation.

18. Wechsler, D., Weschler Intelligence Scale for Children, Fourth Edition, Australian Administration and Scoring Manual. 2003, Marrickville, NSW: Harcourt Assessment.

19. Sheslow, D. and W. Adams, Wide Range Assessment of Memory and Learning. 1990, Wilmington DE: Jastak Associates Inc.

20. Adams, W. and D. Sheslow, Wide Range Assessment of Visual Motor Abilities. 1995, Wilmington: Delaware: Wide Range, Inc.

21. Reitan, R.M. and D. Wolfson, The Halstead Reitan Neuropsychological Test Battery: Theory and clinical interpretation. 1993, Tuscson: Neuropsycchology Press.

22. Ruff, R., RFFT: Ruff Figural Fluency Test: Professional Manual. 1996, Florida: Psychological Assessment Resources.

23. Wilkinson, G.S., The Wide Range Achievement Test Administration Manual. 1993, Wilmington: Delaware: Wide Range, Inc.

24. Hinton-Bayre, A.D., Deriving Reliable Change Statistics from Test–Retest Normative Data: Comparison of Models and Mathematical Expressions. Archives of Clinical Neuropsychology, 2010. 25: p. 244-256.
